# Optimizing early screening for hepatocellular carcinoma: Diagnostic value of ultrasonography combined with serum biomarkers

**DOI:** 10.1097/MD.0000000000043205

**Published:** 2025-07-11

**Authors:** Shijun Yan

**Affiliations:** a Ultrasound Department, Ziyang Yanjiang District People’s Hospital, Ziyang, Sichuan, China.

**Keywords:** early screening, hepatocellular carcinoma, kappa coefficient, ultrasonography

## Abstract

This study assesses the diagnostic performance of ultrasonography combined with serum biomarkers (alpha-fetoprotein [AFP], serum amyloid A [SAA], and C-reactive protein [CRP]) for early detection of hepatocellular carcinoma (HCC) in high-risk individuals, and to evaluate diagnostic agreement using the kappa coefficient to inform optimized screening strategies. A retrospective cohort of 100 high-risk patients screened for HCC between January 2022 and December 2023 was analyzed. Patients were assigned to an experimental group (n = 50; ultrasound + AFP, SAA, CRP) or a control group (n = 50; AFP, SAA, CRP only). Diagnostic performance was evaluated by detection rate, sensitivity, specificity, positive predictive value, negative predictive value, and agreement with contrast-enhanced CT and pathology, using the kappa coefficient (κ). The experimental group exhibited significantly higher detection rate (76.0% vs 50.0%, *P* = .01), sensitivity (88.4% vs 64.1%, *P* = .01), specificity (92.0% vs 78.0%, *P* = .04), positive predictive value (95.0% vs 78.1%, *P* = .02), and negative predictive value (82.1% vs 64.0%, *P* = .03). The kappa coefficient indicated good diagnostic agreement in the experimental group (κ = 0.81) and moderate agreement in the control group (κ = 0.56). Ultrasonography combined with AFP, SAA, and CRP significantly improves early HCC detection and diagnostic consistency and may serve as a routine screening tool in high-risk populations. Integration with artificial intelligence and elastography may further enhance screening accuracy.

## 1. Introduction

Hepatocellular carcinoma (HCC) is the most common type of primary liver cancer and ranks among the leading causes of cancer-related mortality worldwide. Its incidence is rising, particularly among individuals with chronic liver disease or cirrhosis.^[1]^ Due to its asymptomatic nature in early stages, most cases are diagnosed at intermediate or advanced stages, missing the window for curative intervention.^[2]^

Current guidelines recommend regular imaging and serum surveillance in high-risk populations, such as hepatitis B virus (HBV) carriers and patients with cirrhosis.^[3]^ Ultrasonography remains the first-line tool due to its safety, availability, and reproducibility. However, its sensitivity in detecting early-stage or small lesions is limited by operator skill, equipment quality, and lesion characteristics.^[4]^

To enhance detection efficiency, ultrasound is increasingly being combined with serum biomarkers. Alpha-fetoprotein (AFP) remains the most commonly used serological marker for HCC, but it has limited sensitivity and specificity, especially in AFP-negative patients.^[5]^ Serum amyloid A (SAA) and C-reactive protein (CRP) have shown diagnostic potential in recent studies. SAA, in particular, has shown utility in AFP-negative HCC.^[6]^ while CRP may reflect both inflammatory status and prognosis.^[7]^

Contrast-enhanced ultrasound (CEUS) further improves detection by assessing tumor vascularity, and multiparametric strategies incorporating ultrasound with AFP, SAA, and CRP have demonstrated superior performance.^[8,9]^ Additionally, artificial intelligence (AI)-assisted imaging and elastography may offer future enhancements in screening.^[10]^

We hypothesize that ultrasonography combined with serum biomarkers (AFP, SAA, and CRP) will significantly improve the early detection of HCC in high-risk populations, as compared to serum biomarkers alone. This study evaluates the diagnostic value of this combined approach for early HCC detection and assesses its diagnostic agreement with the gold-standard methods of contrast-enhanced CT and histopathology via the kappa coefficient.

## 2. Materials and methods

This study was approved by the Ethics Committee of Ziyang Yanjiang District People’s Hospital. This retrospective study enrolled 100 high-risk patients who underwent HCC screening at our hospital between January 2022 and December 2023.

### 2.1. Grouping

The patients were assigned to either the experimental group or the control group. In this process, each patient was assigned to one of the 2 groups: the experimental group (n = 50) which received ultrasonography combined with serum biomarkers (AFP, SAA, and CRP), or the control group (n = 50) which received serum biomarker testing (AFP, SAA, and CRP) alone.

### 2.2. Inclusion criteria

1. Age 18 to 75 years.
2. Presence of chronic liver disease (e.g., HBV, HCV, cirrhosis, or alcoholic liver disease).
3. No prior diagnosis of HCC.

### 2.3. Exclusion criteria

1. Previously diagnosed HCC.
2. Presence of other malignancies.
3. Incomplete clinical or imaging data.
4. Inability to complete follow-up.

Ethics approval was obtained from the hospital’s Medical Ethics Committee.

### 2.4. Screening protocol

#### 2.4.1. Ultrasonography

Examinations were performed by associate chief physicians or experienced attending physicians (≥3 years in ultrasound). A high-resolution color Doppler ultrasonography system (LOGIQ E9, GE Healthcare, USA) was used. Patients fasted for ≥ 8 hours before imaging.

Liver scanning was conducted in supine and left lateral positions using transverse, sagittal, and oblique views. Key features documented included echogenicity (hypoechoic, isoechoic, or hyperechoic), lesion margin clarity, intratumoral blood flow (via Doppler), and maximum nodule diameter (mm). Two radiologists independently interpreted the results. Disagreements were resolved by a senior radiologist.

#### 2.4.2. Serum marker testing

Fasting venous blood (5 mL) was collected and promptly processed. AFP was measured using electrochemiluminescence immunoassay, and SAA and CRP levels were assessed via immunoturbidimetric assay. The SAA/CRP ratio was calculated as an inflammatory index. All procedures adhered to manufacturer guidelines and quality control standards.

### 2.5. Gold standard diagnosis

Diagnosis of HCC was based on contrast-enhanced CT and histopathology, following criteria established by the American Association for the Study of Liver Diseases (AASLD). Two senior radiologists independently reviewed the images. Discrepancies were resolved via third-party consultation.

### 2.6. Evaluation metrics

Detection rate: confirmed early HCC cases identified by each method.

Diagnostic performance indicators: sensitivity, specificity, positive predictive value, negative predictive value, false positive rate, and false-negative rate.

Diagnostic agreement: assessed via kappa coefficient (κ), interpreted as: <0.40 = poor, 0.40 to 0.75 = moderate, >0.75 = good consistency.

### 2.7. Statistical analysis

SPSS 26.0 (IBM Corp., Armonk) was used for all analyses. Chi-square or Fisher exact test was used for categorical variables; continuous variables were analyzed using independent samples *t* test. A *P* value < .05 was considered statistically significant.

## 3. Results

### 3.1. Baseline characteristics

The experimental and control groups were comparable in terms of age, sex, HBV status, cirrhosis history, alanine aminotransferase, AFP positivity, and SAA/CRP levels (Table 1).

**Table 1:** Baseline characteristics of participants.

Indicator	Experimental group (n = 50)	Control group (n = 50)	*P* value
Age (yr, mean ± SD)	54.2 ± 8.7	53.9 ± 9.1	.84
Sex (male/female)	34/16	32/18	.68
HBV positivity [n (%)	37 (74.0%)	38 (76.0%)	.82
History of cirrhosis [n (%)	28 (56.0%)	30 (60.0%)	.68
ALT (U/L)	47.8 ± 13.2	45.9 ± 12.7	.52
Elevated AFP [n (%)	30 (60.0%)	28 (56.0%)	.69
Elevated SAA/CRP [n (%)	26 (52.0%)	27 (54.0%)	.84

AFP = alpha-fetoprotein, ALT = alanine aminotransferase, CRP = C-reactive protein, HBV = hepatitis B virus, SAA = serum amyloid A.

### 3.2. Diagnostic performance

**Figure 1.**
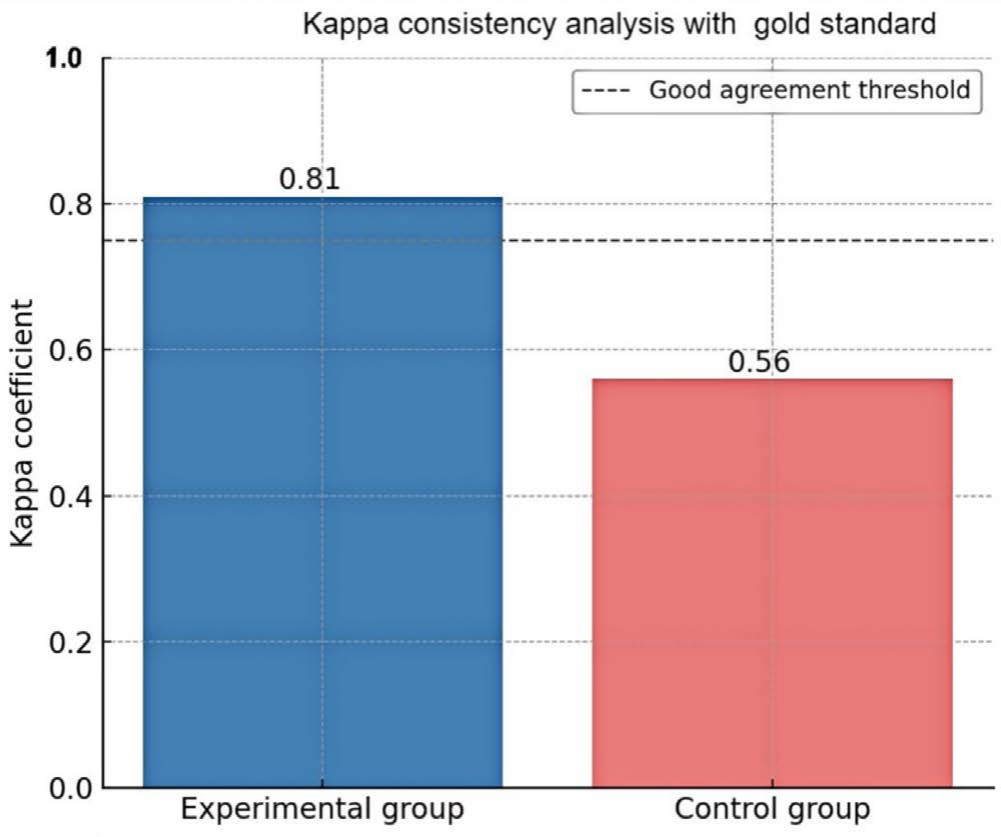
Kappa consistency analysis.

Among 100 high-risk individuals, 82 were diagnosed with early HCC by CT and pathology. The experimental group showed superior performance in all diagnostic indicators (Table 2). Kappa = 0.81 (high concordance), Kappa = 0.56 (moderate concordance) in the experimental group Kappa = 0.56 (moderate concordance) The diagnostic results of the experimental group were in higher concordance with the gold standard, suggesting that the combined screening model is more reliable in actual clinical practice (Fig. 1).

**Table 2:** Diagnostic efficacy analysis of the 2 screening modalities.

Indicator	Experimental group	Control group	*P* value
Detection rate [n (%)]	38 (76.0%)	25 (50.0%)	.01
Sensitivity	88.4% (38/43)	64.1% (25/39)	.01
Specificity	92.0% (23/25)	78.0% (39/50)	.04
PPV	95.0% (38/40)	78.1% (25/32)	.02
NPV	82.1% (23/28)	64.0% (32/50)	.03
FPR	8.0% (2/25)	22.0% (11/50)	.04
FNR	12.0% (5/43)	36.0% (14/39)	.01

FNR = false-negative rate, FPR = false positive rate, NPV = negative predictive value, PPV = positive predictive value.

### 3.3. Ultrasound characteristics of early HCC

**Figure 2.**
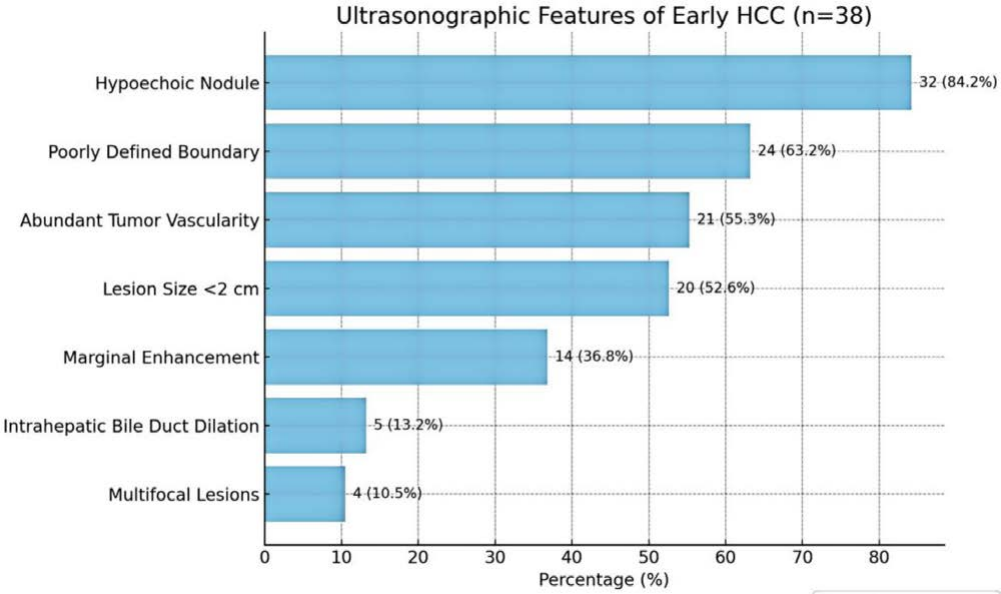
Analysis of the percentage of tumor features found by ultrasound.

**Figure 3.**
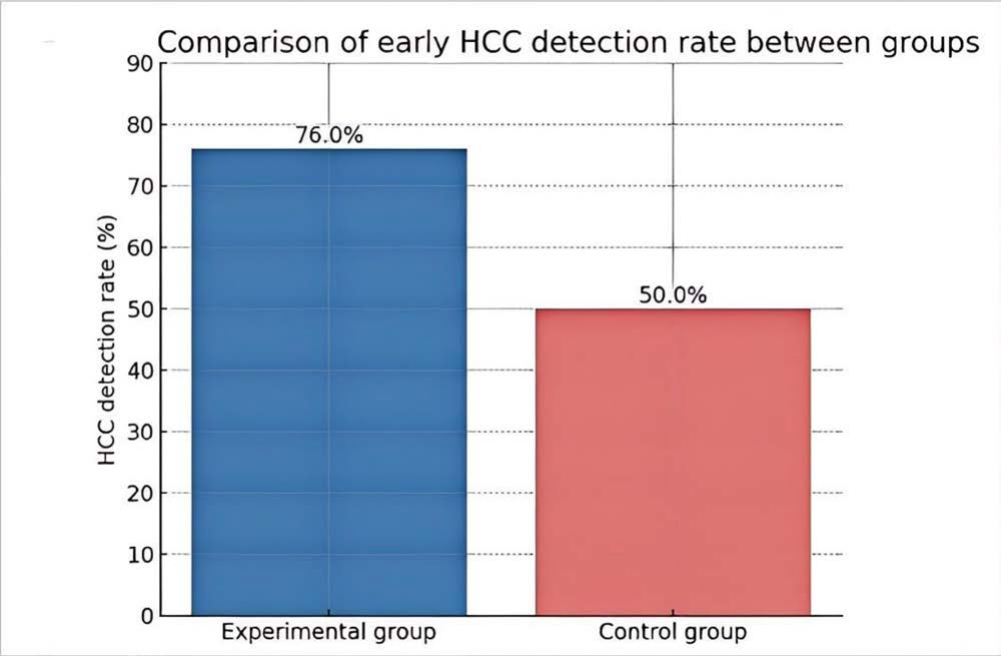
Histogram of confirmed HCC diagnosis rate between groups. HCC = hepatocellular carcinoma.

Table 3 represents the analysis of tumor characteristics detected by ultrasound (n = 38) (Fig. 2). The table summarizes the key ultrasonographic features observed in the study cohort (n = 38). The most prevalent characteristic was hypoechoic nodules (84.2%, 32/38), typically presenting as focal lesions with blurred or mildly defined margins. Poorly defined boundaries were noted in 63.2% (24/38) of cases, characterized by irregular outlines blending into adjacent parenchyma. Over half of the lesions (55.3%, 21/38) exhibited abundant tumor vascularity on Doppler imaging, with prominent arterial signals indicating hypervascularity. Regarding lesion size, 52.6% (20/38) measured < 2 cm, often appearing as solitary nodules, some detected incidentally. A marginal enhancement pattern was observed in 36.8% (14/38), manifesting as a peripheral hyperechoic ring during the arterial phase with progressive enhancement. Less common findings included intrahepatic bile duct dilation (13.2%, 5/38), attributed to localized biliary compression, and multifocal lesions (10.5%, 4/38) distributed across multiple liver lobes. The detection rate of early HCC in the experimental group was significantly higher than that in the control group, indicating that the methods or interventions adopted in the experimental group might be more effective in the early detection of HCC (Fig. 3).

**Table 3:** Ultrasonographic features of early HCC (n = 38).

Feature	n (%)	Description
Hypoechoic nodule	32 (84.2%)	Focal hypoechoic with blurred or slightly defined borders.
Poorly defined boundary	24 (63.2%)	Nodule is irregular in outline and blends into surrounding parenchyma.
Abundant tumor vascularity	21 (55.3%)	Doppler suggests that the tumor has an increased blood supply, and the arterial signal is obvious.
Lesion size < 2 cm	20 (52.6%)	Mostly solitary, some are detected asymptomatically.
Marginal enhancement	14 (36.8%)	Peripheral hyperechoic ring in the arterial phase, with enhancement in the enhancement phase.
Intrahepatic bile duct dilation	5 (13.2%)	Localized compression of the biliary tract.
Multifocal lesions	4 (10.5%)	Multiple nodular foci in different liver lobes.

HCC = hepatocellular carcinoma.

## 4. Discussion

This study demonstrates that ultrasound combined with AFP, SAA, and CRP significantly improves the early detection of HCC in high-risk populations compared to serum markers alone. The combined approach yielded superior sensitivity, specificity, and diagnostic agreement with the reference standard.^[11]^

Ultrasonography is a noninvasive, accessible, and cost-effective imaging modality that has long been employed in HCC surveillance. However, its sensitivity is limited, particularly in detecting small or early-stage lesions, due to influencing factors such as operator expertise, lesion location, and liver echotexture. Our findings reinforced this limitation: the false-negative rate in the control group was markedly higher than in the combined testing group. Recent studies have sought to address these shortcomings by integrating imaging with biomarker-based screening to enhance early detection performance.^[12]^

AFP has been widely used as a traditional biomarker for HCC; however, its poor sensitivity in early-stage disease has been well documented. Some patients with confirmed HCC may still exhibit normal AFP levels, leading to a substantial risk of missed diagnosis.^[13]^ To overcome this limitation, novel biomarkers such as SAA and CRP have received increasing attention. SAA is often significantly elevated in HCC patients and shows diagnostic value particularly in AFP-negative individuals, while CRP serves as both an inflammatory marker and a potential prognostic indicator in liver cancer.^[7]^ Our findings further confirmed that the combination of these 3 biomarkers yielded improved sensitivity and specificity, consistent with prior studies.^[14]^

CEUS, a technique that enhances lesion visualization by assessing intralesional perfusion, can further improve the diagnostic sensitivity for early HCC by revealing vascular patterns and boundary characteristics.^[15]^ Although CEUS was not comprehensively applied in the present study, previous literature suggests that combining CEUS with conventional ultrasound could enhance screening accuracy, offering a valuable direction for future research.^[10]^

Nonetheless, this study has several limitations. The retrospective, single-center design and relatively small sample size introduce the potential for selection bias and limit generalizability.^[16]^ Additionally, ultrasonographic performance is highly operator-dependent, and the variability in scanning quality may affect diagnostic consistency. Therefore, future multicenter prospective studies with standardized ultrasound training protocols are warranted. Lastly, with the ongoing development of AI in medical image analysis, future integration of AI-assisted interpretation with ultrasonography may further enhance screening accuracy and consistency.

Overall, our study supports the use of ultrasound in combination with AFP, SAA, and CRP as an effective early screening strategy for HCC in high-risk populations. This approach can significantly improve the detection rate and diagnostic concordance. In future clinical practice, larger-scale validation and incorporation of advanced technologies such as AI and elastography should be pursued to optimize early HCC detection and improve patient outcomes.

## 5. Conclusion

In conclusion, ultrasonography combined with AFP, SAA, and CRP significantly improves early detection of hepatocellular carcinoma in high-risk populations. This combined approach enhances diagnostic sensitivity, specificity, and agreement with the gold-standard methods. The integration of AI-assisted technologies and elastography may further optimize screening accuracy and clinical outcomes.

## Author contributions

**Conceptualization:** Shijun Yan.

**Data curation:** Shijun Yan.

**Investigation:** Shijun Yan.

**Methodology:** Shijun Yan.

**Supervision:** Shijun Yan.

**Visualization:** Shijun Yan.

**Writing – original draft:** Shijun Yan.

**Writing – review & editing:** Shijun Yan.
